# Inference of transcriptional regulation using gene expression data from the bovine and human genomes

**DOI:** 10.1186/1471-2164-8-265

**Published:** 2007-08-03

**Authors:** Amonida Zadissa, John C McEwan, Chris M Brown

**Affiliations:** 1 Biochemistry Department, University of Otago, PO Box 56, Dunedin, New Zealand; 2 AgResearch, Invermay Agricultural Centre, Mosgiel, Private Bag 50034, New Zealand

## Abstract

**Background:**

Gene expression is in part regulated by sequences in promoters that bind transcription factors. Thus, co-expressed genes may have shared sequence motifs representing putative transcription factor binding sites (TFBSs). However, for agriculturally important animals the genomic sequence is often incomplete. The more complete human genome may be able to be used for this prediction by taking advantage of the expected evolutionary conservation in TFBSs between the species.

**Results:**

A method of *de novo *TFBS prediction based on MEME was implemented, tested, and validated on a muscle-specific dataset.

Muscle specific expression data from EST library analysis from cattle was used to predict sets of genes whose expression was enriched in muscle and cardiac tissues. The upstream 1500 bases from calculated orthologous genes were extracted from the human reference set. A set of common motifs were discovered in these promoters. Slightly over one third of these motifs were identified as known TFBSs including known muscle specific binding sites. This analysis also predicted several highly statistically significantly overrepresented sites that may be novel TFBS.

An independent analysis of the equivalent bovine genomic sequences was also done, this gave less detailed results than the human analysis due to both the quality of orthologue prediction and assembly in promoter regions. However, the most common motifs could be detected in both sets.

**Conclusion:**

Using promoter sequences from human genes is a useful approach when studying gene expression in species with limited or non-existing genomic sequence. As the bovine genome becomes better annotated it can in turn serve as the reference genome for other agriculturally important ruminants, such as sheep, goat and deer.

## Background

To date, many evolutionary studies have focused on conservation of protein coding sequences between species, however, gene expression patterns across species can also be used for evolutionary studies [1]. The focus of this study is on the proximal promoter [2]. Proximal promoters consist of a heterogeneous collection of smaller regulatory elements including transcription factor binding sites (TFBSs). TFBSs are short DNA sequences which bind transcription factors that modulate the level of expression of their cognate gene(s) [3]. Many stimulatory TFBSs are positioned near the transcription start site (TSS), within the proximal promoter region [4]. In some cases, co-regulated genes or tissue-specific genes contain a common set of TFBSs and are controlled by same transcription factors. Analysis of promoter regions is one of the major approaches in understanding the transcriptional regulatory mechanisms. Hence, by identifying the binding sites in the promoters, the pattern of transcriptional regulation may be inferred [5]. Tissue specific promoters, such as those examined in this study often have a single dominant peak and clustering of sequence specific factor binding sites around the TSS [6,7].

The region analysed in this study is 1500 bases prior to the TSS. Deletion assays of the region 1000 bases prior to the TSS of 45 ENCODE promoters [4] indicated positional preference, relative to the TSS, for elements that contribute negatively and positively to the promoter activity. Negative elements resided between -1000 to -500 bp upstream of the TSS, while the positive elements were placed closer to the TSS, between -350 to -40 bp upstream of the TSS in 55% of the genes tested [4]. These conclusions supported previous analyses of functional regions in promoter sequences where it has been established that most (91%) of promoters spanning 550 bp upstream of the TSS had significant transcriptional activity, indicating that the region contains active binding sites [8]. This study does not examine cis-regulatory elements, particularly in enhancers and silencers outside this region [9].

Co-expressed genes can be determined by a number of techniques, such as microarray experiments or EST profiling. These methods usually examine a subset of genes in the genome. However, identifying common TFBSs in these genes allows one to infer the mechanism of co-regulation, establishing genome-wide frequencies of the TFBSs, and detect additional co-regulated genes not present on the array or detected in the original EST library. This approach also has the major benefit that it is sequence-based and is less dependent on prior knowledge on which many other methods of interpreting genomic studies depend. The current challenge is to extend this technique from well-characterised model species, such as human, to those with limited genomic annotation information, such as cattle.

In order to test the feasibility of using the human genome as a reference set for motif prediction, we used a set of bovine and human expression data from cardiac tissue. Initially, we used the homologous human genes and identified a set of motifs common in the promoter regions of the genes. To determine which transcription factors might bind to the predicted motifs and regulate the genes, the motifs were compared with previously described TFBSs and their best hit among known binding sites was identified. These results were then compared with the orthologous bovine promoter sequences where available, applying the same procedure. Finally, we combined and contrasted the various approaches and data sets and examined the underlying substructure of the results using clustering of motif frequencies in the promoter sequences.

We propose and test a general method to deduce regulatory motifs in promoter regions of mammalian species with restricted genomic sequence by using a well-characterised reference genome.

## Results

### Analysis of a *de novo *motif prediction and identification method

Common motifs in promoter sequences were detected using the motif prediction programme MEME [10]. In a recent survey of tools for promoter motif prediction MEME performed well among the thirteen different tools that were assessed [11]. Sequences were analysed on both strands for common *cis*-regulatory elements of length 8 – 12 bp. The predicted elements were subsequently compared to the TFBS databases TRANSFAC [12] and JASPAR [13], for identification of the motifs as potential regulatory elements. For each predicted motif, a series of matches were produced, each representing the best hit from their corresponding length category by a scoring mechanism (see Methods). To determine significant matches, a permutation test was also performed.

**Figure 1:**
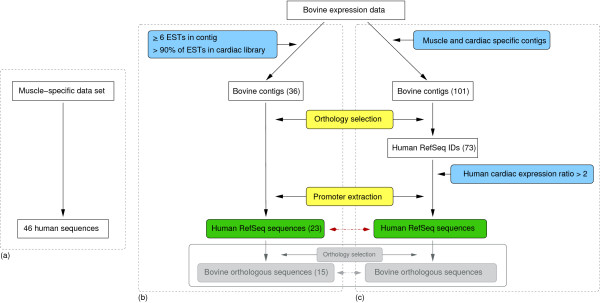
**Flowchart of the data selection**. The diagram shows the three parallel analyses performed for the study. (a) The human subset of the muscle-specific training set curated by Wasserman and Fickett was used for validating the methodology [14] (46 sequences, group a). (b) Bovine cardiac tissue specific expression (group b). Bovine contigs in all libraries were subjected to the following criteria: contigs should consist of a minimum number of 6 ESTs and greater than 90% of those ESTs should be present in the bovine cardiac library. The EST contigs were later passed through the orthology selection where their human RefSeqs were identified. Promoter regions of the obtained RefSeqs were then extracted and examined for common regulatory motifs. Group b is composed of the resulting 23 human sequences. (c) Bovine and human cardiac tissue specific expression (group c). Bovine contigs from four muscle and cardiac tissue libraries were compared to the human genome as described above. The obtained human RefSeqs were subsequently scanned for high expression in human cardiac tissue (log ratio ≤ 2). Group c consists of these 25 human sequences. There are eight common sequences predicted by both methods (b and c) and thus the combined set has 40 sequences. MEME motifs in the combined group were compared (red arrow). The additional analyses done using the bovine orthologues of the human genes in the two data sets are highlighted in the grey box. Common steps in (b) and (c) are coloured yellow. Selection steps are represented by blue boxes.

The approach of *de novo *motif prediction and identification of these motifs as TFBSs was validated using the well-characterised and well-adopted muscle-specific regulatory elements collection and the five related TFBSs described by Wasserman and Fickett [14]. The human subset of this collection comprising 46 sequences and the five matrices acted as the training set (Figure 1a, group a).

**Figure 2:**
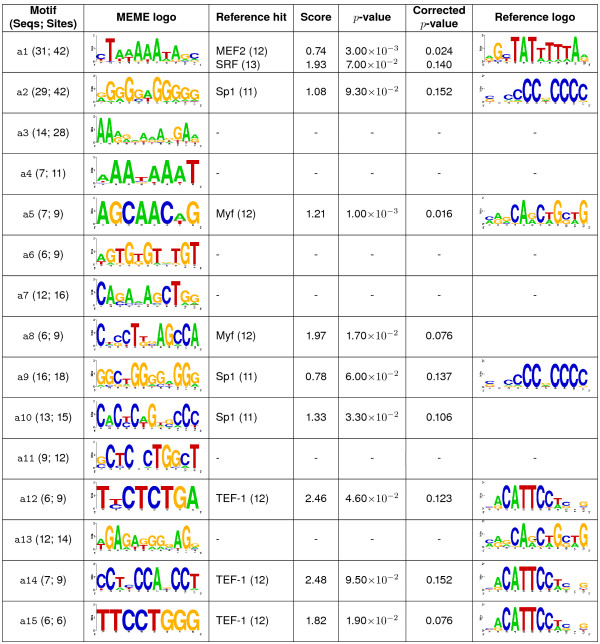
**Identification of expected motifs in the muscle specific data set (group a)**. Sequences in the muscle-specific gene set were examined with MEME for *de novo *prediction of common motifs. The top 15 motifs were then compared to expected muscle TFBSs described in [14]. The motifs are displayed along with the number of sequences containing each motif and the number of sites observed in all sequences in column 1. Sequence logos for the predicted motif and the expected TFBS are displayed, as are the calculated dissimilarity scores (*S*) and *p*-values (*P*) for each comparison. Orientation of the predicted motifs may be reverse to the reference motif, as we consider both strands when predicting the motifs. Motifs marked with (†) have no similar hits in the random sets.

Comparison between matrices for the predicted motifs (Figure 2) in human muscle specific set (a) and those for the binding sites previously determined identified three of the five sites (Figure 2). This indicated that the process had correctly predicted the majority of the matrices. The best match to motif a1 was myocyte enhancer factor 2 (MEF2) with a dissimilarity score (*S*) of 0.74 and *p*-value (*P*) of 3.0 × 10^-3^. Motifs a2, a9 and a10 all matched Sp1, with a9 having the lowest score. The best binding site for the Myf transcription factor was motif a5 with a score of 1.21 and *p*-value of 1.0 × 10^-3^. Motif a8 also matched to Myf but with a higher score of 1.96 (P = 1.7 × 10^-2^). Matches to TEF-1 and SRF were not significant (Figure 2).

The predicted motifs of the muscle specific set were also compared to the TRANSFAC and JASPAR databases. Several motifs had significant matches, with dissimilarity scores below the selected threshold of 1.3, and *p*-values less than 0.05 (Table 1). Comparison of the predicted MEME motifs with the entries in the two databases again identified the first motif (a1) as a MEF2 binding site. Motif a2, although identified as a binding site for the Churchill domain containing 1 protein (CHCH_01), is highly similar to the binding site for the Sp1 transcription factor [15]. Structurally, the Churchill site is similar to the binding site for the Sp1 factor and hence the a2 motif may be an Sp1 binding site. The TRANSFAC SP1_Q6 and SP1_Q2_01 matrices, both representing binding sites for the ubiquitous Sp1 transcription factor, match to a2 with low scores (S < 1), but non-significant *p*-values (Table 1). The a5 motif was identified as a binding site for the Myf factor in JASPAR, confirming the previous annotation of this motif.

**Table 1:** Summary of the identified promoter motifs in Wasserman-Fickett muscle genes of group a

**Motif (Seqs; Sites)**	**Best hit**	**Score **(*S*)	*p***-value **(*P*)	**Corrected ***P*
a1 (42; 31)	MEF2_Q6_01 (12)	0.30	0.00	0.00
	MEF2* (10)	0.68	5.00 × 10^-3^	0.03
a2 (42; 29)	CHCH_01 (6)	0.99	3.60 × 10^-2^	0.09
	SP1_Q6 (13)	0.90	3.70 × 10^-1^	0.37
	SP1_Q2_01 (10)	0.96	3.64 × 10^-1^	0.37
a4 (11; 7)	POU6F1_01 (11)	0.77	2.00 × 10^-2^	0.07
a5 (7; 9)	Myf* (12)	1.21	3.00 × 10^-3^	0.04
a15 (6; 6)	TEL2_Q6 (10)	0.19	0.00	0.00
	ETS_Q4 (12)	0.80	0.00	0.00

Sequences in the muscle-specific gene set were examined with MEME for *de novo *prediction of motifs. The resulting motifs were subsequently compared to entries in the TRANSFAC and JASPAR databases for identification. For each identified motif, the dissimilarity score (*S*) and the *p*-value (*P*) are shown. Corrected *p*-value for each match is based on an estimated FDR of 10%. Matches to the JASPAR database are marked by (*).

Motifs a4 and a15 had significant hits to known TFBS which were different to the expected binding sites. The a4 motif matched the complementary strand to POU6F1_01. The corresponding factor for this matrix is the POU domain, class 6, transcription factor 1. The gene encoding this protein has been shown to be expressed in muscle tissues and is involved in transcriptional regulation of several genes in cardiac tissue [16]. The a15 motif had a high similarity to TEL2_Q6 and its related matrix ETS_Q4, both belonging to the v-ets erythroblastosis virus E26 oncogene gene family.

None of the remaining motifs had matches that met the selection criteria and, therefore, they may represent novel regulatory elements. To estimate false positive rates one thousand randomisations of the original 46 sequences were done and motifs predicted. Among the original set of predicted motifs, seven had no similar hits in the 1000 randomisation sets (indicated in Figure 2) [see Additional file 1]. Four of these are novel a3, a7, a13, although a7 has some similarity to the MYOGNF1_01 site, the myogenin binding site.

Screening all 24030 equivalent RefSeq promoters using MAST gave the genome-wide frequencies of the predicted motifs. Motif a2 is the most frequent among the promoters (4847 occurrences) [see Additional file 2]. The other four motifs similar to known sites, that is a1, a4, a5 and a15, had less than 500 occurrences in the promoters, suggesting that they may be restricted to a subset of genes.

Comparison of the *de novo *predicted motifs in the muscle specific set (a) with three sets of known TFBSs demonstrates that the methodology adopted for the analysis found both known and novel motifs. Five of the 14 unique motifs (36%) could be identified as resembling known TFBSs. The most significant novel motifs were a3 and a7 (Figure 2). These two motifs were common in the dataset with 9 and 16 occurrences but absent in the control randomisation sets [see Additional file 1].

#### Analysis of genes expressed in bovine cardiac cDNA libraries and homologous human genes (group b)

This group of human genes serves as a model set of data representing a genome for which expression data are available but there is limited genomic sequence. Bovine expression data, based on EST sequences were analysed for cardiac-specific expression using two independent methods (Figure 1b and 1c). One method exclusively employed the bovine EST frequency in each tissue library (group b, bovine tissue specific; Table 2), while the other used human cardiac expression data in addition to the bovine data (group c, human cardiac filter; Table 3).

**Table 2:** Human genes in group b and their bovine orthologues

**Human RefSeq ID**	**Gene name**	**Gene description**	**Bovine orthologue Ensembl ID**
NM_000256	MYBPC3	Myosin binding protein C	ENSBTAG00000021707
NM_000257	MYH7	Myosin, heavy polypeptide 7, beta	-
NM_000258	MYL3	Myosin, light polypeptide 3, alkali	-
NM_000363	TNNI3	Troponin I type 3	ENSBTAG00000006424
NM_000432	MYL2	Myosin, light polypeptide 2, regulatory, slow	ENSBTAG00000018369
NM_001001432	TNNT2	Troponin T type 2	ENSBTAG00000006381
NM_001014833	PAK4	p21(CDKN1A)-activated kinase 4	ENSBTAG00000013958
NM_001031729	FRMD5	FERM domain containing 5	ENSBTAG00000017216
NM_001093	ACACB	Aetyl-Coenzyme A carboxylase beta	-
NM_001257	CDH13	Cadherin 13, H-cadherin	-
NM_001995	ACSL1	Acyl-CoA synthetase long-chain family member 1	ENSBTAG00000004344
NM_002471	MYH6	Myosin, heavy polypeptide 6, alpha	-
NM_002536	OATL1	Ornithine aminotransferase-like 1	ENSBTAG00000009288
NM_003827	NAPA	N-ethylmaleimide-sensitive factor attachment protein, alpha	ENSBTAG00000004127
NM_014424	HSPB7	Heat shock 27 kDa protein family, member 7	-
NM_015346	ZFYVE26	Zinc finger, FYVE domain containing 26	ENSBTAG00000014334
NM_015710	GLTSCR2	Glioma tumor suppressor candidate region gene 2	ENSBTAG00000021192
NM_018083	ZNF358	Zinc finger protein 358	ENSBTAG00000013747
NM_058174	COL6A2	Collagen, type VI, alpha 2	ENSBTAG00000019269
NM_130386	COLEC12	Collectin sub-family member 12	ENSBTAG00000007705
NM_153610	CMYA5	Cardiomyopathy associated 5	-
NM_173802	MGC50559	Hypothetical protein MGC50559	ENSBTAG00000000877
NM_194293	CMYA1	Cardiomyopathy associated 1	-

**Table 3:** Human genes in group c

**Human RefSeq ID**	**Gene name**	**Gene description**	**Expr. ratio**
NM_003280	TNNC1	Troponin C type 1, slow	11.183
NM_000257*	MYH7	Myosin, heavy polypeptide 7, beta	10.689
NM_003476	CSRP3	Cysteine and glycine-rich protein 3	9.511
NM_021223	MYL7	Myosin, light polypeptide 7, regulatory	8.883
NM_002471*	MYH6	Alpha myosin heavy chain	8.811
NM_000258*	MYL3	Myosin, light polypeptide 3, alkali	8.517
NM_000432*	MYL2	Myosin light chain 2	8.033
NM_000363*	TNNI3	Troponin I type 3	7.946
NM_005368	MB	Myoglobin	6.732
NM_001001432*	TNNT2	Troponin T type 2	6.492
NM_005159	ACTC	Actin, alpha, cardiac	6.473
NM_014424*	HSPB7	Heat shock 27 kDa protein family, member 7	6.215
NM_198060	NRAP	Nebulin-related anchoring protein	5.058
NM_020707	GUP1	GUP1 glycerol uptake/transporter homolog	5.057
NM_004165	RRAD	Ras-related associated with diabetes	4.527
NM_194293*	CMYA1	Cardiomyopathy associated 1	4.152
NM_001151	SLC25A4	Solute carrier family 25	3.926
NM_001312	CRIP2	Cysteine-rich protein 2	3.823
NM_016581	SITPEC	Evolutionarily conserved signaling intermediate in Toll pathway	3.342
NM_016150	ASB2	Ankyrin repeat and SOCS box-containing 2	3.075
NM_001098	ACO2	Aconitase 2	3.005
NM_133378	TTN	Titin	2.927
NM_016599	MYOZ2	Myozenin 2	2.583
NM_002667	PLN	Phospholamban	2.248
NM_020376	PNPLA2	Patatin-like phospholipase domain containing 2	2.110

Asterisks (*) indicate common human genes with group b.

The EST sequences from 48 selected bovine tissue libraries were assembled into contigs and those contigs containing six or more ESTs were retained. These contigs were then analysed for cardiac tissue specific expression and 36 were selected. Putative human homologues for the contigs were obtained through BLASTN analysis [17], by comparing the bovine contig consensus sequences to the human RefSeq database (RefSeq release as of 02/09/2005), giving rise to 23 unique human RefSeq genes (Figure 1b, Table 2). The genes include well-known muscle-specific genes encoding sarcomeric or sarcomeric-associated proteins, such as Troponin T type 2 (*TNNT2*) and Troponin I type 3 (*TNNI3*), myosin binding protein C (*MYBPC3*) and other myosin-related genes.

A segment of 1500 bp upstream of the TSS for each of the genes was analysed for common *cis*-regulatory elements. This region is expected to contain most of the binding sites that are involved in transcription initiation [7,8].

**Figure 3:**
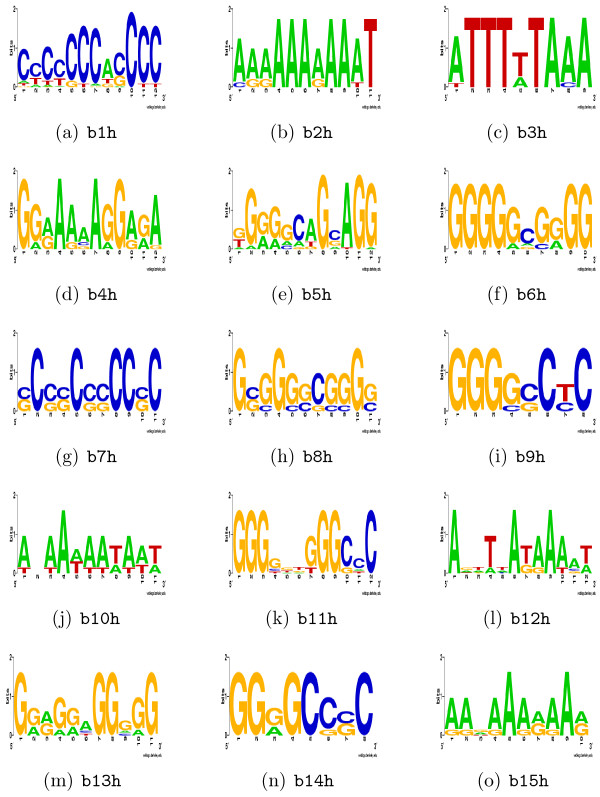
**Sequence logos of predicted MEME motifs in the 23 human RefSeq promoters of group b**. Sequence logos of the top 15 motifs predicted by MEME in the 23 human RefSeq genes in group b. The suffix h denotes "human", for example b1 h is the first predicted motif in the human promoters in group b.

Table 4 along with Figure 3 summarises the motifs predicted by MEME in the 23 human promoters and their best matches in TRANSFAC and JASPAR. There were significant similarities between several of the motifs, and could be collapsed into six groups [see Additional file 3] indicating that they may represent variations of the same element (TFBS). Binding sites for the Sp1 and MEF2 factors were identified among the predicted motifs, b1 h and b6 h respectively. Other motifs identified include E47_01 (motif b5 h), a binding site for the E47 factor. This protein, along with E12, are splice variants from the E2A gene and can interact with the muscle-specific transcription factor MyoD by dimerising with this protein [18]. Motif b8 h was identified as a binding site for the AP2 protein. The AP2 factor is known to be involved in regulation of muscle development [19]. Motif b11 h had significant similarity to MZF 5–13, which corresponds to the second DNA binding domain of myeloid zinc finger gene 1 (*MZF1*). Its role in the regulation of muscle genes has not previously been reported. Detailed information about the motifs and their hits are available in [see Additional file 3]. Motifs b2 h and b4 h were the most significant novel motifs.

**Table 4:** Summary of the identified promoter motifs in the 23 human RefSeq genes of group b

**Motif (Seqs; Sites)**	**Best hit**	**Score **(*S*)	*p***-value **(*P*)	**Corrected ***P*
b1 h (17; 45)	SP1_Q2_01 (10)	0.87	5.71 × 10^-1^	0.62
	SP1_Q4_01 (13)	0.98	7.82 × 10^-1^	0.78
b3 h (7; 10)	AMEF2_Q6 (18)	0.87	4.00 × 10^-3^	0.06
b5 h (15; 29)	E47_01 (15)	0.99	1.10 × 10^-2^	0.06
b8 h (4; 6)	AP2_Q3 (16)	0.78	9.00 × 10^-3^	0.06
b11 h (6; 6)	MZF 5–13* (10)	0.67	5.00 × 10^-3^	0.08

Promoter sequences for the 23 human genes were examined with MEME for prediction of motifs common to the group. The resulting motifs were then compared to entries in the TRANSFAC and JASPAR databases for identification. For each identified motif the dissimilarity score (*S*) and the *p*-value (*P*) are shown. Corrected *p*-value for each match is based on an estimated FDR of 10%. Matches to the JASPAR database are marked by (*). Sequence logos for the matrices are given in Figure 3.

#### Analysis of genes expressed in bovine muscle cDNA libraries and human cardiac tissue (group c)

A group of 101 bovine EST contigs [see Additional file 4] expressed specifically in four muscle and cardiac libraries was selected (Figure 1c).

Comparison with the human genome identified 73 unique human RefSeq genes [see Additional file 5]. These were later filtered for expression in human cardiac tissue using microarray expression ratios [20] extracted from the UCSC genome browser. Of the 73 human sequences, 25 met the cutoff of an expression ratio of > 2 in cardiac tissue. Therefore, this approach also employs gene expression information from the reference genome. The methodology is useful when the equivalent information in the original genome is not available.

**Figure 4:**
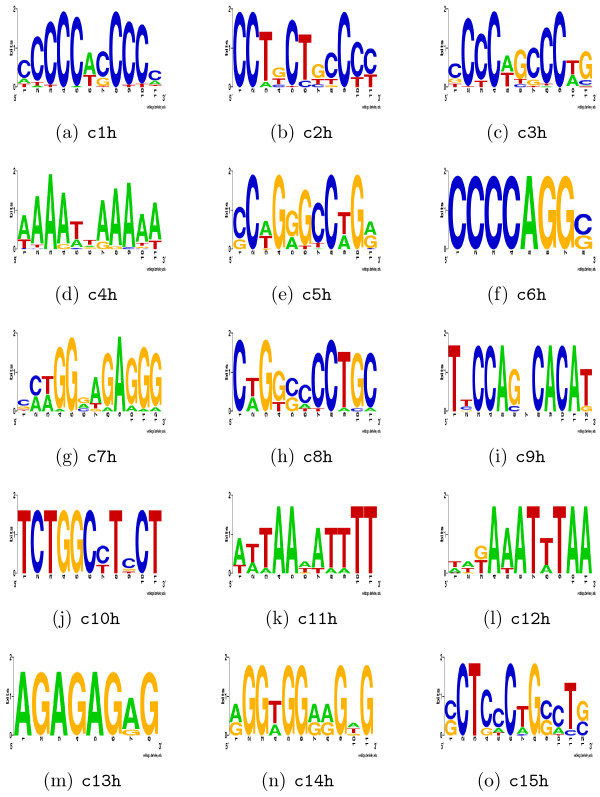
**Sequence logos of predicted MEME motifs in promoters of 25 human RefSeq cardiac genes of group c**. Sequence logos of the top 15 motifs predicted by MEME in the 25 human RefSeq genes in group c. The resulting motifs were compared to the TRANSFAC and JASPAR databases for detecting known binding sites. The suffix h denotes "human".

Promoter sequences for the above 25 genes were analysed as described before. The motif prediction in promoter regions of the human genes identified 15 distinct motifs (Figure 4) with no similarities between the motifs. Nine of the 15 predicted motifs had matches to known TFBSs (Table 5). The Sp1 binding site was again detected among the motifs (c1 h). None of the Sp1 site matches to c1 h were significant by the *p*-value criteria, although the dissimilarity scores for the matches clearly indicate that the site may indeed be a binding site for the Sp1 factor. Other motifs identified in this group are the TATA-box element (TBP_Q6 in TRANSFAC) present in promoters and a binding site for the GATA4 transcription factor. The GATA4 protein, a cardiac tissue-specific transcription factor, is expressed in early cardiac progenitor cells [21]. The c4 h, c9 h, c14 h and c15 h were identified PAX4, PAX, EGRF4 and MAZ binding sites, respectively [see Additional file 6].

**Table 5:** Identified motifs in promoters of 25 human RefSeq cardiac genes in group c

**Motif (Seqs; Sites)**	**Best hit**	**Score (***S***)**	*p***-value (***P***)**	**Corrected ***P*
c1 h (20; 49)	SP1_Q2_01 (10)	0.86	4.54 × 10^-1^	0.48
	SP1_Q6 (13)	0.99	6.02 × 10^-1^	0.60
c2 h (15; 36)	GATA4_Q3 (12)	0.96	2.40 × 10^-2^	0.07
c4 h (11; 30)	Pax-4* (30)	0.95	0.00	0.00
c9 h (6; 7)	PAX_Q6 (11)	0.44	3.00 × 10^-3^	0.03
c10 h (5; 6)	RP58_01 (12)	0.90	1.80 × 10^-2^	0.07
c11 h (3; 8)	TBP_Q6 (7)	0.73	1.70 × 10^-2^	0.07
c12 h (12; 8)	TBP_Q6 (7)	0.71	4.00 × 10^-2^	0.08
c14 h (8; 12)	NGFIC_01 (12)	0.89	4.00 × 10^-3^	0.03
c15 h (12; 17)	MAZ_Q6 (8)	0.66	2.70 × 10^-2^	0.07

The sequences were analysed by MEME to predict common motifs. The resulting motifs were compared to the TRANSFAC and JASPAR databases for identifying known binding sites. The dissimilarity score (*S*) and *p*-value (*P*) for each comparison are shown. Corrected *p*-value for each match is based on an estimated FDR of 10%. Matches to the JASPAR database are marked by (*). Sequence logos for the matrices are given in Figure 4.

Analysis of the motifs predicted in group c shows the presence of several potentially muscle-specific regulatory elements found in the promoters of human genes. Compared to genes in group b, there was far less redundancy in the predicted motifs, which may be due to slightly larger data sets and also the expression specificity of the selected data, as expression array information may more accurately predict expression than EST profiling.

### Frequency of the predicted motifs in the combined cardiac specific promoters

The unique MEME motifs, seven predicted in group b (Figure 3) and all the 15 motifs in group c (Figure 4), were combined together (indicated with red arrow in Figure 1). Similar motifs were identified and collapsed using MAST [22].

**Figure 5:**
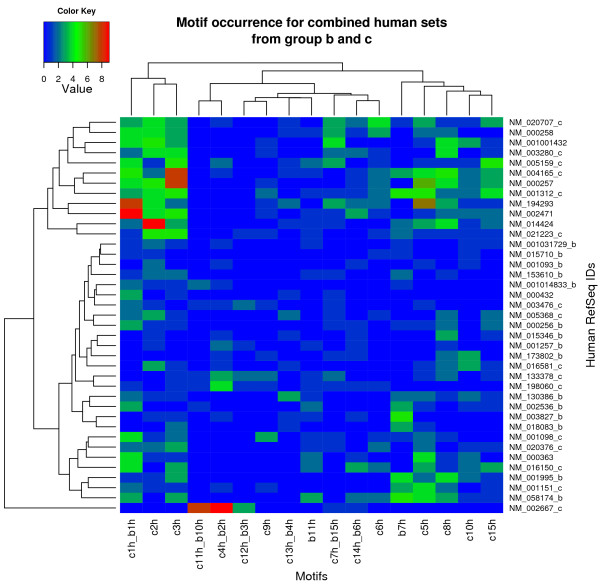
**Colour map of combined motifs observed in all human promoters from groups b and c**. The map displays the motif occurrences in the two combined human sets. Columns represent unique motifs (r < 0.6) in the merged set and rows are the RefSeqs from the two sets. Similar motifs from the two sets are denoted and displayed with the names of the motifs joined, e.g. the first motif is common in both sets as motif c1 h in group c and b1 h in group b. Promoters unique to the individual sets are suffixed by the name of the corresponding group. If several motifs from a data set are similar, only the first one is shown. Sequence for Myozenin 2 (NM_016599) was omitted as the combined motif *e*-value was not significant in this sequence (*e*-value > 10).

The sequences from cardiac groups b and c were combined and eight common sequences were identified, resulting in 40 unique sequences. Predicted motifs from the two groups were then clustered based on their frequency in all the promoters (Figure 5). This was done to reveal information which may not be obvious from the global motif analysis, for example whether presence and frequency levels of specific motifs can be related to the functions of the corresponding genes. Figure 5 shows a colour map of the non-redundant motifs and their frequency in the promoter sequences. Of the seven unique motifs in group b, five were in common with group c (r > 0.6). A distinct group of promoter sequences has the highest frequencies of the c1 h_b1 h, c2 h and c3 h motifs (top left in Figure 5). The first two motifs were identified as TFBSs for the Sp1 and GATA4 transcription factors, respectively. The c3 h motif had no matches and may represent a novel motif related to expression of the genes. The promoter group containing these motifs consists of 12 sequences, including several of the sarcomeric genes described earlier. This suggests a likely role of the motifs in relation to the common function of the genes. The same group of promoters also display a high frequency of other motifs (top right in Figure 5). These latter motifs also form a subcluster and their presence in the same group of promoters implies possible transcriptional regulation in a modular fashion whereby the presence of several transcription factors is required for activation of transcription. The two subclusters of motifs are also present in another group of promoters (lower part of Figure 5) but at a less pronounced frequency. These genes have a number of functions, such as energy pathways, cell communication and cell growth. The promoter sequence of the phospholamban gene (*PLN*) does not cluster with any other sequence and has three distinct motifs at high rates. These motifs were identified as TFBSs for the TBP (c11 h and c12 h), MEF2 (b3 h) and homeodomain of PAX4 (c4 h) factors.

An examination of the motif frequency patterns in the promoters shows that five of the seven unique motifs (71%) are in common between the two sets [see Additional file 7]. This is an indication that using the inherent bovine EST frequencies in tissue libraries is sufficient for the analysis of regulatory patterns.

The 40 promoters predicted by either methods (b and c) to be cardiac specific were examined for overlap with CpG islands [23], only 11 overlapped. None of the eight promoters predicted by both methods had CpG islands within the 1500 bases upstream of the TSS only one had a TATA box within 50 bases. This is consistent with our finding of c1 h_b1 h/GC/Sp1 sites in these promoters (Figure 5).

### Analysis of bovine genome sequences

The bovine genome is the first of the artidactyl genomes to be sequenced. The Btau2.0, 6.2 fold coverage assembly comprises 1.7 Gb with only 24% in contigs > 1000 bases. The small contig size creates problems for extraction of regions upstream of the TSS or first coding exon. Orthologous bovine promoters from the 23 human RefSeqs in group b, were retrieved using Ensembl's gene orthology criteria [24]. Fifteen orthologues could be retrieved (Table 1).

The 15 sequences were analysed as before. Of the six unique motifs, three were in common with motifs predicted in their human orthologues corresponding to AP2 (b11b group, b15b, b11b) and MEF2 (b2b group) [see Additional file 8]. Two novel motifs (b1b, and b3b) occurred frequently with 19 and 25 occurrences, in 8 and 11 of 15 sequences [see Additional file 7].

## Discussion

A common problem in agricultural science is incomplete genomic sequence for the species of interest, and therefore limited or no access to the promoter sequences. The proposed solution is to identify orthologous genes in related species that have sequenced genomes and extract the relevant promoter regions. These promoters can then be used to search for common regulatory motifs. The approach used here was to examine the promoter regions of co-expressed genes for shared motifs. Such a method can identify both known and novel TFBSs, it can also potentially identify genes that may be co-expressed but that have not been measured as part of the experiment.

In this paper we present a simple approach for deducing regulatory information in mammalian genomes with restricted sequence by using a reference genome. We evaluate this approach using an independent data set. We used MEME [22] for predicting motifs in promoter sequences of the genes. MEME performed well in a recent assessment of tools employed for prediction of TFBSs [11] and offers a flexible set of parameters.

Wasserman and Fickett [14] compiled and analysed a group of experimentally verified muscle regulatory regions and discovered the presence of five specific binding sites in these sequences. This human subset (group a) of this collection was used as the training set to evaluate the proposed approach. Promoter analysis of sequences in the training set identified three of the five expected binding sites for muscle-specific transcription factors. We identified MEF2, Sp1 and Myf binding sites significantly in the set. The SRF binding site also matched to the same motif identified as a MEF2 site (a1). This result suggests that MEME may be clustering these two binding sites together due to the similarity at the centre of both sites. The randomisation of the training data also supported this observation. The composition, that is number of sequences and sites that contribute to the structure of a motif, of several low ranking motifs, such as a7, a9, a10 and a13, were very distinct from motifs generated from the random sequences. Hence, the approach used was to examine all 15 predicted motifs regardless of their MEME reported *e*-values in the subsequent analyses. We conclude from this initial comparison that our approach and selection criteria identified the majority of the motifs present.

The predicted motifs in group a were also compared to known TFBSs in TRANSFAC [12] and JASPAR [13]. These databases are the most comprehensive collection of known TFBSs available. The TRANSFAC database is the largest available set of known TFBSs. The core matrices of many profiles have also been computationally extended – introducing potential redundancy into the data. Many of the longer binding sites are contributing to non-specific matches. The size of the JASPAR database is much smaller than TRANSFAC but it consists of experimentally verified and highly curated profiles. Overall in this study more of the TRANSFAC hits had *p*-values < 0.05 compared to those in JASPAR.

The MEF2 binding site was identified. The *MEF2 *gene family members are expressed during early embryogenesis and throughout the developing myocardium and are well-characterised for their involvement in muscle differentiation [25-27]. The conserved DNA binding domain in these regulators recognises an AT-rich consensus sequence, present in the regulatory regions of many muscle-specific genes [28,29]. The MEF2 motifs in the TRANSFAC and JASPAR databases are largely based on *in vitro *SELEX data and differ slightly from each other and from that in [14].

Sp1 like motifs were also be identified, including the CHCH_01 motif in group a, that is a partial motif of the Sp1 binding site. The POU6F1 binding site identified in the set may be a novel muscle element. POU6F1 is a member of the homeobox protein family, involved in developmental processes, and has been shown to be expressed in muscle tissue [16]. Another potentially novel factor for transcriptional regulation of muscle genes is TEL2, a member of the ETS family. However, this same motif matched the ETS-1 motif itself, which has been shown to be involved in cardiac morphogenesis [30,31] and the TEF-1 motif in JASPAR also matched the same motif, indicating a similar composition in the two motifs.

Once we had evaluated our method, we subsequently utilised bovine cDNA expression data for the study. Contigs resulting from assembling the initial ESTs were analysed for muscle and cardiac tissue-specific expression using two methods. The first method used only the bovine EST frequency per library data. The second method employed both bovine and human expression data. Fourteen motifs (47%) of the initially 30 predicted motifs in the human promoters were identified as known TFBSs. Comparison of the unique motifs detected in the human promoters of groups b and c show that 71% in group b are in common between the two sets. These results indicate that even though the genes in the two sets were selected partially independently from each other, they produce similar results.

Once promoter sequences sharing common regulatory elements were identified, the combination of motifs present in the promoters could then be used to determine additional substructure within the results using clustering based on the number TFBSs present in the promoters. This suggests that the number and order of TFBSs present in a promoter encodes information that can be easily extracted from the results of this approach. The eight common promoters contained multiple Sp1 like sites (Figure 5) and generally lacked CpG islands and TATA boxes near to the TSS. Promoters with these characteristics generally had a single dominant peak of TSS [6] in other tissues, but potential alternative promoter starts were not examined in this study.

For the bovine genome two assemblies are currently in use (Btau2.0) used here and Btau3.1. The latest assembly has increased the size of the contigs to 61% > 1000 (rather than 24%) [32]. However, this is still much lower than that of human at 94% > 5000. Therefore the assembly in regulatory regions, particularly the link between these and the coding regions remains much better for the human genome, and the human genome will remain a useful reference.

However, the current method suffers from some caveats. It does not use all available information, such as co-localisation and order of the TFBSs. Additionally, the method is not well-suited for determining gene regulation in tissues not shared between species, such as the rumen in artiodactyl mammals. This will therefore impose restrictions on the kind of analyses that may be performed. This barrier will, however, be overcome with a comprehensive annotation of bovine genome.

In the latest version of MEME, it is now possible to compare the produced PFMs to known motifs in JASPAR [33], a procedure similar to this study. It produces similar results but is also limited by the incompleteness of the JASPAR database as found here.

Examining the evolutionary conservation of the motifs can give further information about individual motifs and their inferred involvement in transcriptional regulation. Using available tools for phylogenetic footprinting studies, such as multi-species sequence alignments from public databases, e.g. the UCSC or Ensembl genome browsers, can aid in this task. The recently developed PhyMe [34] and PhyloGibbs [35] programmes address this proposed approach, using motif over-representation coupled with phylogenetic comparison to calculate significance of the predicted motifs. These will become more powerful as the number of genomes sequenced increases and the coverage and quality of their assembly improve.

## Conclusion

By comparing two methods, both initially based on bovine EST data, we show that using human promoter regions as a reference platform in interpreting ruminant expression studies is a viable solution for the analysis of gene regulation patterns in the bovine genome.

The proposed method is simple and easy to implement with existing software and is robust when sufficient co-expressed (co-regulated) sequences can be identified. Finally, as the bovine genome becomes better annotated, it can serve as an interim platform for many other agriculturally important animals, such as sheep and goat, until their genome sequences become available.

## Methods

### Collection of data sets

In this paper, we analyse three data sets. The first set consists of regulatory regions involved in muscle-specific gene expression. This group was compiled by Wasserman and Fickett to study transcription factors associated with skeletal muscle gene regulation [14]. They constructed a set of five position frequency matrices based on binding sites for MEF2, Myf, SRF, TEF-1 and Sp1 transcription factors which were found to bind within these sequences. The regulatory regions for the human subset and the matrices served as the training set to validate our approach. The sequences used were analysed with the MEME programme [10] (v3, release date: 02/04/2002) to identify common motifs. Frequency matrices of predicted motifs were subsequently compared to the known binding sites above using the matrix similarity implementation of mutual entropy described below. This group is referred to as group a in the paper. The collection of elements was retrieved from [36].

The second and third data sets (groups b and c) were based on bovine contigs assembled using bovine sequences – a collection of sequences from AgResearch bovine EST libraries along with all bovine cDNAs submitted to NCBI sequence repositories at the time (September 2005). The contigs were generated from the ESTs using CAP3 [37] and resulted in 6223 contigs, each containing ≥ 6 ESTs. In order to distinguish cardiac-specific genes, the contigs were subjected to the following criterion: more than 90% of the ESTs in the contig should be present in the bovine cardiac library. The contigs were then used for retrieving their human homologues. Annotation of the sequences was performed using their corresponding human RefSeq sequence (RefSeq release as of 02/09/2005). The BLASTN [17] sequence analysis was employed to identify sequence homology and each bovine contig was annotated with the best human RefSeq homologue. If several bovine contigs matched the same human gene, the gene was reported only once, resulting in a list of unique human RefSeq genes. The human RefSeq sequences comprised the second data set for the analysis, group b. In parallel to this approach, human orthologues of contigs in four bovine muscle libraries (NCBI dbEST ID numbers: 18993, 18997, 18987 and 19022) were examined for their expression levels in human cardiac tissue using publicly available human gene expression results. The human RefSeqs were submitted to the University of California-Santa Cruz (UCSC) Human Gene sorter [38] using the GNF Gene Expression Atlas 2 [20]. Genes with an expression log ratio of 2 or higher in heart tissue were selected. These human genes comprised group c. Figure 1 displays a diagram of the different approaches and the data selection procedure.

### Promoter extraction

A region of 1500 bp upstream of the transcription start site for all the human RefSeq genes was extracted using the UCSC human genome browser [23]. The sequences were masked for repetitive elements during the retrieval process. Intersect with CpG island prediction (cpgIslandExt) was done using the UCSC table browser. The promoters of the orthologous bovine genes were retrieved from Ensembl.

### Motif discovery

All data sets, groups a – c, were separately examined to identify common and potentially functional elements present in the promoter regions using MEME. Based on the assumption that transcription factor binding sites would likely consist of small and highly conserved motifs, the programme was set to output the top 15 motifs from each set, with motif length ranging between 8 and 12 bases long. We also allowed for each motif to occur any number of times in a single sequence. The minimum and maximum number of sites were allowed to vary between 6 and 50 respectively. The reverse complement strand of each sequence was also considered in the analysis. Sequence logos for all the motifs were generated using WebLogo [39]. Using the MAST programme, promoter sequences for all human RefSeq genes were screened for the presence of all unique motifs from each data set.

### Motif clustering

The promoters of the human genes in groups b and c were combined together, as were the motifs predicted in the two sets, for identification of all motifs shared between the sets. Duplicated sequences were removed. Any motifs with a Pearson's correlation coefficient > 0.6 were identified and collapsed. The motif frequencies in the different promoters were analysed using hierarchical clustering in R [40], where the dissimilarities between the frequencies were taken into account. The resulting matrix was then used to generate a colour map. The same procedure was also applied to the bovine promoter sequences from groups b and c.

### Identification of known motifs

To test for known biological relevance of the MEME motifs, the transcription factor databases TRANSFAC v8.1 [12] and JASPAR [13] were used. The flat file for the TRANSFAC database was accessible through a commercial licence while the JASPAR matrix files were freely available from [41]. The database flat files were parsed using the TFBS Perl modules [42] to generate position frequency matrices (PFMs). Only the vertebrate matrices from the databases were employed, resulting in 522 TRANSFAC and 83 JASPAR matrices respectively.

### Matrix similarity

Consider the predicted motifs generated by MEME and the known sites from the TRANSFAC and JASPAR databases. The corrected probability, *p*_*bj*_, of observing nucleotide *b *at position *j *is given by:

pbj=fbj+αbN+∑αb,
 MathType@MTEF@5@5@+=feaafiart1ev1aaatCvAUfKttLearuWrP9MDH5MBPbIqV92AaeXatLxBI9gBaebbnrfifHhDYfgasaacH8akY=wiFfYdH8Gipec8Eeeu0xXdbba9frFj0=OqFfea0dXdd9vqai=hGuQ8kuc9pgc9s8qqaq=dirpe0xb9q8qiLsFr0=vr0=vr0dc8meaabaqaciaacaGaaeqabaqabeGadaaakeaacqWGWbaCdaWgaaWcbaGaemOyaiMaemOAaOgabeaakiabg2da9maalaaabaGaemOzay2aaSbaaSqaaiabdkgaIjabdQgaQbqabaGccqGHRaWkiiGacqWFXoqydaWgaaWcbaGaemOyaigabeaaaOqaaiabd6eaojabgUcaRmaaqaeabaGae8xSde2aaSbaaSqaaiabdkgaIbqabaaabeqab0GaeyyeIuoaaaGccqGGSaalaaa@4258@

where *f*_*bj *_denotes the observed frequency of each base at position *j*, *N *is the total number of sites and *α*_*b *_are constants added to correct for small sample sizes. Given two position weight matrices (PWMs), *M*_1 _and *M*_2_, the relative entropy or dissimilarity score (*S*), also known as the Kullbeck-Liebler distance, of the two PWMs can be calculated as:

S(M1,M2)=∑j=1l∑b∈{A,C,G,T}p1bj⋅ln⁡(p1bjp2bj),
 MathType@MTEF@5@5@+=feaafiart1ev1aaatCvAUfKttLearuWrP9MDH5MBPbIqV92AaeXatLxBI9gBamXvP5wqSXMqHnxAJn0BKvguHDwzZbqegyvzYrwyUfgarqqtubsr4rNCHbGeaGqiA8vkIkVAFgIELiFeLkFeLk=iY=Hhbbf9v8qqaqFr0xc9pk0xbba9q8WqFfeaY=biLkVcLq=JHqVepeea0=as0db9vqpepesP0xe9Fve9Fve9GapdbaqaaeGacaGaaiaabeqaamqadiabaaGcbaGaem4uamLaeiikaGIaemyta00aaSbaaSqaaiabigdaXaqabaGccqGGSaalcqWGnbqtdaWgaaWcbaGaeGOmaidabeaakiabcMcaPiabg2da9maaqahabaWaaabuaeaacqWGWbaCdaWgaaWcbaGaeGymaeJaemOyaiMaemOAaOgabeaakiabgwSixlGbcYgaSjabc6gaUnaabmaabaWaaSaaaeaacqWGWbaCdaWgaaWcbaGaeGymaeJaemOyaiMaemOAaOgabeaaaOqaaiabdchaWnaaBaaaleaacqaIYaGmcqWGIbGycqWGQbGAaeqaaaaaaOGaayjkaiaawMcaaaWcbaGaemOyaiMaeyicI4Saei4EaSNaemyqaeKaeiilaWIaem4qamKaeiilaWIaem4raCKaeiilaWIaemivaqLaeiyFa0habeqdcqGHris5aaWcbaGaemOAaOMaeyypa0JaeGymaedabaGaemiBaWganiabggHiLdGccqGGSaalaaa@7372@

where *M*_1 _and *M*_2 _are assumed to have the same number of columns, *l*. As the comparison is asymmetric, the average between *S*(*M*_1_, *M*_2_) and *S*(*M*_2_, *M*_1_) is selected as the score for comparing two matrices. The dissimilarity score (*S*), ranging between 0 and 6 in this study, is an indication of the degree of dissimilarity between the two matrices – i.e. a value of zero indicates a perfect match while higher values indicate less homology between the two matrices.

Given two sets of PFMs, one comprising the predicted matrices and the other a database of known matrices for TFBSs, a Perl [43] script was used to carry out matrix comparisons – comparing each predicted matrix to all the entries in the database. To compare two PFMs of different lengths, a sliding window with length equal to the shorter PFM was used. At each comparison instant the following criteria has to be satisfied: the length of the comparison window has to be at least six bases long, and the overlapping segment of at least one of the matrices has to have a minimum of 60% information content in total. The final score for a comparison is normalised by dividing by the length of the comparing window. As transcription factors have the ability to bind to the DNA on either strand, the Perl script also tests the reverse complement of the predicted MEME matrices. The database matrices are divided into categories depending on their length. Best matches from each length category are reported, resulting in a series of hits for each of the MEME matrices. The final best match for each predicted matrix was selected from these by using random permutations of each MEME matrix to estimate statistical significance (see below).

### Permutation analysis

In order to evaluate the statistical significance of the obtained matrix matches, each MEME matrix was randomly permuted 1000 times to obtain a *p*-value. The permutations re-sort the base composition of the matrix while keeping other associations such as the GC content and the depth of the matrix, i.e. the number of sites, unchanged. The false discovery rate (FDR) of the procedure was estimated by adjusting the *p*-values resulting from the permutations for the number of hypotheses tested, i.e. 15 matrix comparisons. The p.adjust programme in the R stats package [40] was used for the purpose. Sequences in the training set were shuffled 1000 times to generate 1000 random sequence sets. These were subsequently examined by MEME where in total 15000 motifs were generated. The motifs were used to obtain background distributions for number of sequences and number of sites that comprised the motifs.

## Authors' contributions

AZ was involved in the conception of the project, undertook the data collection and analysis, the computer programming and contributed the majority of the manuscript. JCM and CMB were involved in the conception of the project, advised on data collection and analysis, and wrote and edited portions of the manuscript. All authors read and approved the final manuscript.

## Availability and requirements

Software is available at .

## Supplementary Material

Additional file 1 Predicted motifs in training set. This file displays all 15 motifs detected in the muscle-specific training set (group a). Click here for file

Additional file 2 Genome frequency of motifs in group a. This file contains a table of genome-wide frequencies of the predicted motifs in group a. Click here for file

Additional file 3 Predicted motifs in the 23 human genes (group b). This file shows the 15 motifs detected in the promoter regions of 23 human genes in group b. Click here for file

Additional file 4 Bovine contig sequences in FASTA format.: The bovine contigs that were used in the study are given in this file. Click here for file

Additional file 5 Initial human RefSeq genes for group c. This table gives a list of the 73 human RefSeq genes that were identified when the 101 bovine contigs in group c were compared to the human genome. Click here for file

Additional file 6 Predicted motifs in the 25 human genes (group c). This file contains the 15 motifs detected in the promoter regions of the 25 human genes in group c. Click here for file

Additional file 7 Comparison of motifs predicted in all data sets. The unique motifs (r < 0.6) in each data set from the two groups, b and c, were cross-compared with motifs in all other sets. Predicted unique motifs in each set are on the diagonal. The off-diagonal values displays the number of highly correlated motifs in each compared pair of groups. Click here for file

Additional file 8 Predicted motifs in the 15 bovine genes (group b). This file shows the 15 motifs detected in the promoter regions of the 15 bovine genes in group b. Click here for file

## References

[B1] Khaitovich P, Pääbo S, Weiss G (2005). Toward a neutral evolutionary model of gene expression. Genetics.

[B2] Maston GA, Evans SK, Green MR (2006). Transcriptional Regulatory Elements in the Human Genome. Annu Rev Genomics Hum Genet.

[B3] Bejerano G, Pheasant M, Makunin I, Stephen S, Kent WJ, Mattick JS, Haussler D (2004). Ultraconserved elements in the human genome. Science.

[B4] Cooper SJ, Trinklein ND, Anton ED, Nguyen L, Myers RM (2006). Comprehensive analysis of transcriptional promoter structure and function in 1% of the human genome. Genome Res.

[B5] Xie X, Lu J, Kulbokas EJ, Golub TR, Mootha V, Lindblad-Toh K, Lander ES, Kellis M (2005). Systematic discovery of regulatory motifs in human promoters and 3' UTRs by comparison of several mammals. Nature.

[B6] Carninci P, Kasukawa T, Katayama S, Gough J, Frith MC, Maeda N, Oyama R, Ravasi T, Lenhard B, Wells C, Kodzius R, Shimokawa K, Bajic VB, Brenner SE, Batalov S, Forrest AR, Zavolan M, Davis MJ, Wilming LG, Aidinis V, Allen JE, FANTOM Consortium, RIKEN Genome Exploration Research Group, Genome Science Group (Genome Network Project Core Group) m (2005). The transcriptional landscape of the mammalian genome. Science.

[B7] ENCODE Project Consortium (2007). Identification and analysis of functional elements in 1% of the human genome by the ENCODE pilot project. Nature.

[B8] Trinklein ND, Aldred SJ, Saldanha AJ, Myers RM (2003). Identification and functional analysis of human transcriptional promoters. Genome Res.

[B9] Pennacchio LA, Ahituv N, Moses AM, Prabhakar S, Nobrega MA, Shoukry M, Minovitsky S, Dubchak I, Holt A, Lewis KD, Plajzer-Frick I, Akiyama J, De Val S, Afzal V, Black BL, Couronne O, Eisen MB, Visel A, Rubin EM (2006). In vivo enhancer analysis of human conserved non-coding sequences. Nature.

[B10] Bailey T, Elkan C (1994). Fitting a mixture model by expectation maximization to discover motifs in biopolymers. Proc Int Conf Intell Syst Mol Biol.

[B11] Tompa M, Li N, Bailey T, Church G, De Moor B, Eskin E, Favorov A, Frith M, Fu Y, Kent W, Makeev V, Mironov A, Noble W, Pavesi G, Pesole G, Régnier M, Simonis N, Sinha S, Thijs G, van Helden J, Vandenbogaert M, Weng Z, Workman C, Ye C, Zhu Z (2005). Assessing computational tools for the discovery of transcription factor binding sites. Nat Biotechnol.

[B12] Matys V, Fricke E, Geffers R, Gössling E, Haubrock M, Hehl R, Hornischer K, Karas D, Kel A, Kel-Margoulis O, Kloos D, Land S, Lewicki-Potapov B, Michael H, Münch R, Reuter I, Rotert S, Saxel H, Scheer M, Thiele S, Wingender E (2003). TRANSFAC: transcriptional regulation, from patterns to profiles. Nucleic Acids Res.

[B13] Sandelin A, Alkema W, Engström P, Wasserman W, Lenhard B (2004). JASPAR: an open-access database for eukaryotic transcription factor binding profiles. Nucleic Acids Res.

[B14] Wasserman W, Fickett J (1998). Identification of regulatory regions which confer muscle-specific gene expression. J Mol Biol.

[B15] Zhao C, Meng A (2005). Sp1-like transcription factors are regulators of embryonic development in vertebrates. Dev Growth Differ.

[B16] Wey E, Lyons GE, Schäfer BW (1994). A human POU domain gene, mPOU, is expressed in developing brain and specific adult tissues. Eur J Biochem.

[B17] Altschul S, Gish W, Miller W, Myers E, Lipman D (1990). Basic local alignment search tool. J Mol Biol.

[B18] Lingbeck JM, Trausch-Azar JS, Ciechanover A, Schwartz AL (2005). E12 and E47 modulate cellular localization and proteasome-mediated degradation of MyoD and Id1. Oncogene.

[B19] Bragança J, Eloranta JJ, Bamforth SD, Ibbitt JC, Hurst HC, Bhattacharya S (2003). Physical and functional interactions among AP-2 transcription factors, p300/CREB-binding protein, and CITED2. J Biol Chem.

[B20] Su A, Wiltshire T, Batalov S, Lapp H, Ching K, Block D, Zhang J, Soden R, Hayakawa M, Kreiman G, Cooke M, Walker J, Hogenesch J (2004). A gene atlas of the mouse and human protein-encoding transcriptomes. Proc Natl Acad Sci U S A.

[B21] Peterkin T, Gibson A, Loose M, Patient R (2005). The roles of GATA-4, -5 and -6 in vertebrate heart development. Semin Cell Dev Biol.

[B22] Bailey T, Gribskov M (1998). Combining evidence using p-values: application to sequence homology searches. Bioinformatics.

[B23] UCSC genome browser. http://genome.ucsc.edu/.

[B24] Birney E, Andrews D, Caccamo M, Chen Y, Clarke L, Coates G, Cox T, Cunningham F, Curwen V, Cutts T, Down T, Durbin R, Fernandez-Suarez XM, Flicek P, Gräf S, Hammond M, Herrero J, Howe K, Iyer V, Jekosch K, Kähäri A, Kasprzyk A, Keefe D, Kokocinski F, Kulesha E, London D, Longden I, Melsopp C, Meidl P, Overduin B, Parker A, Proctor G, Prlic A, Rae M, Rios D, Redmond S, Schuster M, Sealy I, Searle S, Severin J, Slater G, Smedley D, Smith J, Stabenau A, Stalker J, Trevanion S, Ureta-Vidal A, Vogel J, White S, Woodwark C, Hubbard TJ (2006). Ensembl 2006. Nucleic Acids Res.

[B25] Black BL, Olson EN (1998). Transcriptional control of muscle development by myocyte enhancer factor-2 (MEF2) proteins. Annu Rev Cell Dev Biol.

[B26] Anderson JP, Dodou E, Heidt AB, Val SJD, Jaehnig EJ, Greene SB, Olson EN, Black BL (2004). HRC is a direct transcriptional target of MEF2 during cardiac, skeletal, and arterial smooth muscle development in vivo. Mol Cell Biol.

[B27] Molkentin JD, Firulli AB, Black BL, Martin JF, Hustad CM, Copeland N, Jenkins N, Lyons G, Olson EN (1996). MEF2B is a potent transactivator expressed in early myogenic lineages. Mol Cell Biol.

[B28] Edmondson DG, Lyons GE, Martin JF, Olson EN (1994). Mef2 gene expression marks the cardiac and skeletal muscle lineages during mouse embryogenesis. Development.

[B29] Lin Q, Schwarz J, Bucana C, Olson EN (1997). Control of mouse cardiac morphogenesis and myogenesis by transcription factor MEF2C. Science.

[B30] Macías D, Pérez-Pomares JM, García-Garrido L, Carmona R, Muñnoz-Chápuli R (1998). Immunoreactivity of the ets-1 transcription factor correlates with areas of epithelial-mesenchymal transition in the developing avian heart. Anat Embryol (Berl).

[B31] Lie-Venema H, de Groot ACG, van Empel LJP, Boot MJ, Kerkdijk H, de Kant E, DeRuiter MC (2003). Ets-1 and Ets-2 transcription factors are essential for normal coronary and myocardial development in chicken embryos. Circ Res.

[B32] Bos taurus genome: Statistics – Build 3.1 (based on Btau_3.1). http://www.ncbi.nlm.nih.gov/mapview/stats/BuildStats.cgi?taxid=9913&build=3&ver=1.

[B33] Bailey TL, Williams N, Misleh C, Li WW (2006). MEME: discovering and analyzing DNA and protein sequence motifs. Nucleic Acids Res.

[B34] Sinha S, Blanchette M, Tompa M (2004). PhyME: a probabilistic algorithm for finding motifs in sets of orthologous sequences. BMC Bioinformatics.

[B35] Siddharthan R, Siggia ED, Nimwegen Ev (2005). PhyloGibbs: a Gibbs sampling motif finder that incorporates phylogeny. PLoS Comput Biol.

[B36] Repository of functional regulatory elements. http://pipmaker.bx.psu.edu/mousegroup/Reg_annotations/.

[B37] Huang X, Madan A (1999). CAP3: A DNA sequence assembly program. Genome Res.

[B38] Karolchik D, Baertsch R, Diekhans M, Furey T, Hinrichs A, Lu Y, Roskin K, Schwartz M, Sugnet C, Thomas D, Weber R, Haussler D, Kent W (2003). The UCSC Genome Browser Database. Nucleic Acids Res.

[B39] Crooks GE, Hon G, Chandonia JM, Brenner SE (2004). WebLogo: a sequence logo generator. Genome Res.

[B40] R Development Core Team, R Foundation for Statistical Computing (2006). R: A Language and Environment for Statistical Computing.

[B41] JASPAR matrix sites. http://jaspar.cgb.ki.se/DOWNLOAD/SITES/.

[B42] Lenhard B, Wasserman W (2002). TFBS: Computational framework for transcription factor binding site analysis. Bioinformatics.

[B43] Perl programming language. http://www.perl.com.

