# Supplementary material for: Inference of transcriptional regulation using gene expression data from the bovine and human genomes
Source: BMC Genomics. 2007 Aug 3;8:265. doi: 10.1186/1471-2164-8-265 (PMC1978505; doi:10.1186/1471-2164-8-265)
Supplement: Additional file 1 — Predicted motifs in training set. This file displays all 15 motifs detected in the muscle-specific training set (group a). [file 1471-2164-8-265-S1.pdf]

Additional file 1 (Zadissa et al.)

Identified motifs in the muscle-specific evaluation set compared to TRANSFAC and JASPAR matrices. The motifs are designated as a1, a2, etc. The values in brackets indicate the number of sequences containing each motif and the number of sites observed in all sequences. Sequence logos for the predicted motif and the expected TFBS are displayed, as are the calculated dissimilarity scores (S) and p-values (P) for each comparison (S_{cutoff} = 1.3, P_{cutoff} = 0.05). Randomised seq and site frequencies display the number of sequences and sites based on 1000 randomisation of the original set. The corrected p-value were calculated using the values from the permutation runs for each pair-wise matrix comparison. Sequence logos for the predicted motifs are displayed, as are the scores (S) and p-values for the best matches in TRANSFAC and JASPAR. Motifs marked with (†) have no equivalents among the randomised sets.

Motif (Seqs; Sites)	Randomised seq. freq.	Randomised site freq.	Sequence logo	TRANSFAC hit	Score	p-value	Corrected p-value	JASPAR hit	Score	p-value	Corrected p-value
a1 (31; 42)†	0.000	0.000	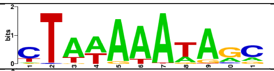	MEF2_Q6_01 (12)	0.30	0.000	0.000	MEF2 (10)	0.68	0.005	0.030
a2 (29; 42)†	0.000	0.000	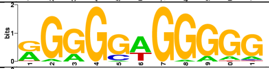	CHCH_01 (6)<br>SP1_Q6 (13)<br>SP1_Q2_01 (10)	0.99<br>0.90<br>0.96	0.036<br>0.370<br>0.364	0.093<br>0.370<br>0.370	MZF_1-4 (6)	1.28	0.404	0.433
a3 (14; 28)†	0.000	0.000	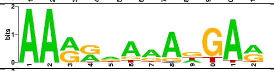	ETS_Q6 (8)	0.97	0.262	0.295	SPI-B (7)	1.55	0.160	0.200
a4 (7; 11)	0.307	0.008	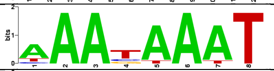	POU6F1_01 (11)	0.77	0.020	0.072	SRY (9)	1.38	0.057	0.143
a5 (7; 9)	0.261	0.032	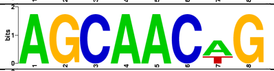	MIF1_01 (18)	1.52	0.032	0.093	Myf (12)	1.21	0.003	0.038
a6 (6; 9)	0.635	0.044	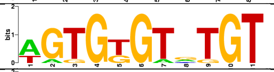	AP2REP_01 (7)	1.56	0.086	0.138	FREAC-3 (8)	2.10	0.087	0.163
a7 (12; 16)†	0.000	0.000	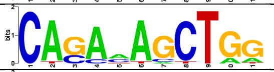	MYOGNF1_01 (29)	1.42	0.003	0.013	deltaEF1 (6)	1.42	0.150	0.200
a8 (6; 9)	0.130	0.034	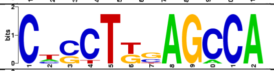	ALPHACP1_01 (11)	1.38	0.089	0.138	deltaEF1 (6)	1.39	0.100	0.167
a9 (16; 18)†	0.000	0.000	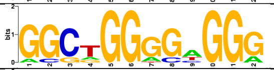	CACBINDINGPROTEIN_Q6 (9)	1.00	0.065	0.130	MZF_1-4 (6)	1.48	0.228	0.263
a10 (13; 15)†	0.000	0.000	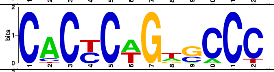	LFA1_Q6 (8)	1.10	0.065	0.130	Androgen (22)	2.06	0.071	0.152
a11 (9; 12)	0.034	0.000	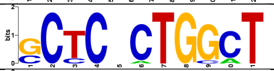	SP1_Q6 (13)	1.60	0.180	0.231	Pax-4 (30)	2.15	0.016	0.060
a12 (6; 9)	0.575	0.029	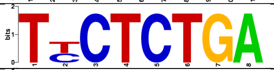	ATF1_Q6 (11)	1.42	0.092	0.138	SPI-B (7)	1.61	0.010	0.050
a13 (12; 14)†	0.000	0.000	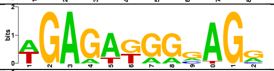	MAZ_Q6 (8)	1.24	0.207	0.248	MZF_1-4 (6)	1.75	0.591	0.591
a14 (7; 9)	0.238	0.015	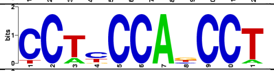	CACBINDINGPROTEIN_Q6 (9)	1.39	0.126	0.174	Staf (20)	1.86	0.117	0.176
a15 (6; 6)	0.538	0.428	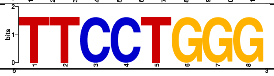	TEL2_Q6 (10)<br>ETS_Q4 (12)	0.19<br>0.80	0.000<br>0.000	0.000<br>0.000	TEF-1 (12)	1.82	0.048	0.143
