# Supplementary material for: Inference of transcriptional regulation using gene expression data from the bovine and human genomes
Source: BMC Genomics. 2007 Aug 3;8:265. doi: 10.1186/1471-2164-8-265 (PMC1978505; doi:10.1186/1471-2164-8-265)
Supplement: Additional file 2 — Genome frequency of motifs in group a. This file contains a table of genome-wide frequencies of the predicted motifs in group a. [file 1471-2164-8-265-S2.pdf]

Additional file 2 (Zadissa et al.)

Frequency of the motifs across the genome using MAST. Motifs with an e-value $>$ 10 were excluded, i.e. motif a9.

**Motif**	a1	a2	a3	a4	a5	a6	a7	a8	a10	a11	a12	a13	a14	a15
**Genome frequency**	452	4851	887	247	295	947	686	990	2056	1174	475	967	1715	385
