# Supplementary material for: Inference of transcriptional regulation using gene expression data from the bovine and human genomes
Source: BMC Genomics. 2007 Aug 3;8:265. doi: 10.1186/1471-2164-8-265 (PMC1978505; doi:10.1186/1471-2164-8-265)
Supplement: Additional file 5 — Initial human RefSeq genes for group c. This table gives a list of the 73 human RefSeq genes that were identified when the 101 bovine contigs in group c were compared to the human genome. [file 1471-2164-8-265-S5.pdf]

Additional file 5 (Zadissa et al.)

**Human RefSeq**	**Gene name**	**Description**	**Bovine Ensembl ID**
NM_000127	EXT1	Exostoses (multiple) 1	ENSBTAG00000006209
NM_000183	HADHB	Hydroxyacyl-Coenzyme A dehydrogenase/3-ketoacyl-Coenzyme A thiolase/enoyl-Coenzyme A hydratase (trifunctional protein), beta subunit	ENSBTAG00000010083
NM_000256	MYBPC3	Myosin binding protein C, cardiac	ENSBTAG00000021707
NM_000257	MYH7	Myosin, heavy polypeptide 7, cardiac muscle, beta	-
NM_000258	MYL3	myosin, light polypeptide 3, alkali; ventricular, skeletal, slow	-
NM_000352	ABCC8	ATP-binding cassette, sub-family C (CFTR/MRP), member 8	-
NM_000363	TNNI3	Troponin I type 3 (cardiac)	ENSBTAG00000006424
NM_000432	MYL2	Myosin, light polypeptide 2, regulatory, cardiac, slow	ENSBTAG00000018369
NM_001001432	TNNT2	Troponin T type 2 (cardiac)	ENSBTAG00000006381
NM_001014832	PAK4	p21(CDKN1A)-activated kinase 4	ENSBTAG00000013958
NM_001031729	FRMD5	FERM domain containing 5	ENSBTAG00000017216
NM_001093	ACACB	Aetyl-Coenzyme A carboxylase beta	-
NM_001098	ACO2	Aconitase 2, mitochondrial	ENSBTAG00000006424
NM_001151	SLC25A4	Solute carrier family 25 (mitochondrial carrier; adenine nucleotide translocator), member 4	ENSBTAG00000013208
NM_001179	ART3	ADP-ribosyltransferase 3	ENSBTAG00000010954
NM_001257	CDH13	Cadherin 13, H-cadherin (heart)	-
NM_001312	CRIP2	Cysteine-rich protein 2	ENSBTAG00000004661
NM_001846	COL4A2	Collagen, type IV, alpha 2	-
NM_001995	ACSL1	Acyl-CoA synthetase long-chain family member 1	ENSBTAG00000004344
NM_002471	MYH6	Myosin, heavy polypeptide 6, cardiac muscle, alpha	-
NM_002493	NDUFB6	NADH dehydrogenase (ubiquinone) 1 beta subcomplex, 6, 17kDa	ENSBTAG00000005907
NM_002536	OATL1	Ornithine aminotransferase-like 1	ENSBTAG00000009288
NM_002541	OGDH	Oxoglutarate (alpha-ketoglutarate) dehydrogenase (lipoamide)	ENSBTAG00000006029
NM_002622	PFDN1	Prefoldin subunit	ENSBTAG00000016596
NM_002667	PLN	Phospholamban	ENSBTAG00000012931
NM_002709	PPP1CB	Protein phosphatase 1, catalytic subunit, beta isoform	ENSBTAG00000012447
NM_003130	SRI	Sorcin	ENSBTAG00000010390
NM_003260	TLE2	Transducin-like enhancer of split 2 (E(sp1) homolog, Drosophila)	ENSBTAG00000001153
NM_003280	TNNC1	Troponin C type 1 (slow)	ENSBTAG00000020336
NM_003476	CSRP3	Cysteine and glycine-rich protein 3 (cardiac LIM protein)	ENSBTAG00000011869
NM_003827	NAPA	N-ethylmaleimide-sensitive factor attachment protein, alpha	ENSBTAG00000004127
NM_004165	RRAD	Ras-related associated with diabetes	ENSBTAG00000013929
NM_004714	DYRK1B	Dual-specificity tyrosine-(Y)-phosphorylation regulated kinase 1B	ENSBTAG00000012509
NM_005159	ACTC	Actin, alpha, cardiac muscle	ENSBTAG00000005714
NM_005368	MB	Myoglobin	ENSBTAG00000005333
NM_005388	PDCL	Phosducin-like	ENSBTAG00000014778

Human RefSeq	Gene name	Description	Bovine Ensembl ID
NM_006185	NUMA1	Nuclear mitotic apparatus protein 1	ENSBTAG00000018449
NM_007286	SYNPO	Synaptopodin	ENSBTAG00000013744
NM_013392	NRBP1	Nuclear receptor binding protein 1	ENSBTAG00000018153
NM_014216	ITPK1	Inositol 1,3,4-triphosphate 5/6 kinase	ENSBTAG00000009845
NM_014222	NDUFA8	NADH dehydrogenase (ubiquinone) 1 alpha subcomplex, 8, 19kDa	ENSBTAG00000004295
NM_014424	HSPB7	Heat shock 27kDa protein family, member 7	-
NM_014268	MAPRE2	microtubule-associated protein, RP/EB family, member 2	ENSBTAG00000007520
NM_015346	ZFYVE26	Zinc finger, FYVE domain containing 26	ENSBTAG00000014334
NM_015415	BRP44	Brain protein 44	ENSBTAG00000020968
NM_015710	GLTSCR2	Glioma tumor suppressor candidate region gene 2	ENSBTAG00000021192
NM_015966	ERGIC3	ERGIC and golgi 3	ENSBTAG00000006670
NM_016150	ASB2	Ankyrin repeat and SOCS box-containing 2	ENSBTAG00000007109
NM_016581	SITPEC	Signaling intermediate in Toll pathway, evolutionarily conserved	ENSBTAG00000015049
NM_016599	MYOZ2	Myozenin 2	ENSBTAG00000002574
NM_018082	POLR3B	Polymerase (RNA) III (DNA directed) polypeptide B	ENSBTAG00000004781
NM_018083	ZNF358	Zinc finger protein 358	ENSBTAG00000013747
NM_020376	PNPLA2	Patatin-like phospholipase domain containing 2	ENSBTAG00000005144
NM_020707	GUP1	GUP1, glycerol uptake/transporter homolog (yeast)	-
NM_021223	MYL7	Myosin, light polypeptide 7, regulatory	ENSBTAG00000002066
NM_022039	FBXW4	F-box and WD-40 domain protein 4	ENSBTAG00000003579
NM_022895	C12orf43	Chromosome 12 open reading frame 43	ENSBTAG00000021796
NM_030633	KIAA1712	KIAA1712	ENSBTAG00000020055
NM_030752	TCP1	T-complex 1	ENSBTAG00000002829
NM_030935	TSC22D4	TSC22 domain family, member 4	ENSBTAG00000015272
NM_032126	C1orf49	Chromosome 1 open reading frame 49	ENSBTAG00000004941
NM_032810	ATAD1	ATPase family, AAA domain containing 1	ENSBTAG00000000806
NM_032982	CASP2	Caspase 2, apoptosis-related cysteine peptidase	ENSBTAG00000018159
NM_058174	COL6A2	Collagen, type VI, alpha 2	ENSBTAG00000019269
NM_130386	COLEC12	Collectin sub-family member 12	ENSBTAG00000007705
NM_133378	TTN	Titin	-
NM_139157	ST5	Suppression of tumorigenicity 5	ENSBTAG00000005356
NM_152783	D2HGDH	D-2-hydroxyglutarate dehydrogenase	ENSBTAG00000002847
NM_153610	CMYA5	Cardiomyopathy associated 5	-
NM_173802	MGC50559	Hypothetical protein MGC50559	ENSBTAG00000000877
NM_181805	PKIG	Protein kinase (cAMP-dependent, catalytic) inhibitor gamma	ENSBTAG00000005275
NM_194293	CMYA1	Cardiomyopathy associated 1	-
NM_198060	NRAP	Nebulin-related anchoring protein	ENSBTAG00000019327
