# Supplementary material for: Inference of transcriptional regulation using gene expression data from the bovine and human genomes
Source: BMC Genomics. 2007 Aug 3;8:265. doi: 10.1186/1471-2164-8-265 (PMC1978505; doi:10.1186/1471-2164-8-265)
Supplement: Additional file 6 — Predicted motifs in the 25 human genes (group c). This file contains the 15 motifs detected in the promoter regions of the 25 human genes in group c. [file 1471-2164-8-265-S6.pdf]

Additional file 6 (Zadissa et al.)

Identified motifs detected in the promoter regions of the 25 human genes in group c. Sequence logos for the predicted motif and the expected TFBS are displayed, as are the calculated dissimilarity scores (S) and p-values (P) for each comparison (S_{cutoff} = 1.3, P_{cutoff} = 0.05).

Motif (Seqs; Sites)	Sequence logo	TRANSFAC hit	Score	p-value	Corrected p-value	JASPAR hit	Score	p-value	Corrected p-value
c1h (20; 49)	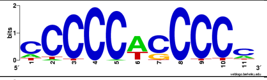	SP1_Q2_01 (10)<br>SP1_Q6 (13)	0.86<br>0.99	0.454<br>0.602	0.484<br>0.602	SP1 (10)	1.32	0.651	0.751
c2h (15; 36)	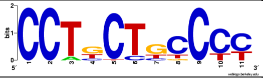	GATA4_Q3 (12)	0.96	0.024	0.072	Gklf (10)	1.20	0.059	0.153
c3h (16; 46)	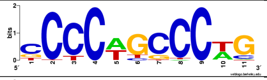	EGR_Q6 (11)	0.96	0.060	0.102	AP2alpha (9)	1.50	0.001	0.004
c4h (11; 30)	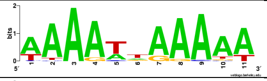	IRF_Q6 (15)	0.86	0.145	0.166	Pax-4 (30)	0.95	0.000	0.000
c5h (16; 28)	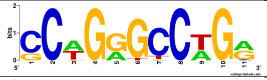	AP2_Q6_01 (13)	1.33	0.092	0.129	AP2alpha (9)	1.76	0.061	0.153
c6h (13; 20)	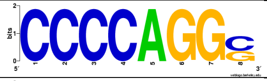	AP2_Q3 (16)	1.50	0.038	0.080	AP2alpha (9)	2.04	0.077	0.165
c7h (18; 25)	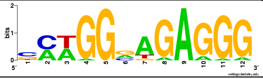	POLY_C (18)	1.37	0.064	0.102	Gklf (10)	1.43	0.116	0.218
c8h (11; 16)	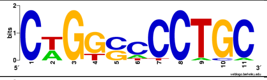	MAZ_Q6 (8)	1.18	0.097	0.129	deltaEF1 (6)	2.28	0.144	0.240
c9h (6; 7)	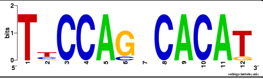	PAX_Q6 (11)	0.44	0.003	0.032	Thing1-E47 (10)	1.42	0.001	0.004
c10h (5; 6)	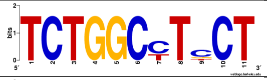	RP58_01 (12)	0.90	0.018	0.072	SP1 (10)	1.76	0.491	0.614
c11h (3; 8)	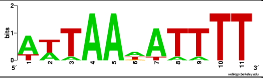	TBP_Q6 (7)	0.73	0.017	0.072	SRY (9)	1.64	0.178	0.267
c12h (12; 8)	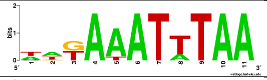	TBP_Q6 (7)	0.73	0.040	0.080	TBP (15)	1.60	0.405	0.552
c13h (6; 6)	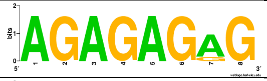	POLY_C (18)	1.89	0.115	0.142	GATA-3 (6)	2.21	0.990	1.000
c14h (8; 12)	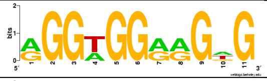	NGFIC_01 (12)	0.89	0.004	0.032	Pax-4 (30)	1.63	0.000	0.000
c15h (12; 17)	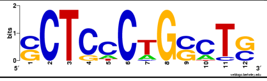	MAZ_Q6 (8)	0.66	0.027	0.072	SP1 (10)	2.17	1.000	1.000
