# Supplementary material for: Inference of transcriptional regulation using gene expression data from the bovine and human genomes
Source: BMC Genomics. 2007 Aug 3;8:265. doi: 10.1186/1471-2164-8-265 (PMC1978505; doi:10.1186/1471-2164-8-265)
Supplement: Additional file 7 — Comparison of motifs predicted in all data sets. The unique motifs (r < 0.6) in each data set from the two groups, b and c, were cross-compared with motifs in all other sets. Predicted unique motifs in each set are on the diagonal. The off-diagonal values displays the number of highly correlated motifs in each compared pair of groups. [file 1471-2164-8-265-S7.pdf]

Additional file 7 (Zadissa et al.)

The unique motifs (r < 0.6) in each data set from the two groups, b and c, were cross–compared with motifs in all other sets. Predicted unique motifs in each set are on the diagonal. The off–diagonal values displays the number of highly correlated motifs in each compared pair of groups.

		group b		group c	
		human	bovine	human	bovine
group b	human	7			
	bovine	4	6		
group c	human	5	1	15	
	bovine	4	3	3	14
