# Supplementary material for: Inference of transcriptional regulation using gene expression data from the bovine and human genomes
Source: BMC Genomics. 2007 Aug 3;8:265. doi: 10.1186/1471-2164-8-265 (PMC1978505; doi:10.1186/1471-2164-8-265)
Supplement: Additional file 3 — Predicted motifs in the 23 human genes (group b). This file shows the 15 motifs detected in the promoter regions of 23 human genes in group b. [file 1471-2164-8-265-S3.pdf]

Additional file 3 (Zadissa et al.)

Identified motifs detected in the promoter regions of 23 human genes in group b. Sequence logos for the predicted motif and the expected TFBS are displayed, as are the calculated dissimilarity scores (S) and p-values (P) for each comparison(S_{cutoff} = 1.3, P_{cutoff} = 0.05). MAST analysis showed high similarity between the motifs, collapsing the 15 motifs in to seven groups. These are: b1h; b2h, b8h, b14h and b15h; b3h and b12h; b4h, b5h, b6h and b13h; b7h, b9h and b14h; b10h and b11h.

Motif (Seqs; Sites)	Sequence logo	TRANSFAC hit	Score	p-value	Corrected p-value	JASPAR hit	Score	p-value	Corrected p-value
b1h (17; 45)	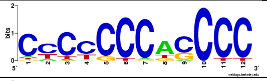	SP1_Q2_01 (10)<br>SP1_Q4_01 (13)	0.87<br>0.98	0.571<br>0.782	0.618<br>0.782	p50 (11)	1.08	0.104	0.365
b2h (9; 9)	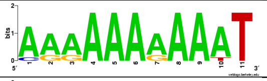	PAX4_04 (30)	1.36	0.146	0.328	Pax-4 (30)	1.75	0.432	0.665
b3h (7; 10)	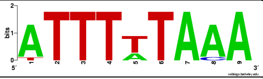	AMEF2_Q6 (18)	0.87	0.004	0.059	MEF2 (10)	1.21	0.033	0.248
b4h (12; 12)	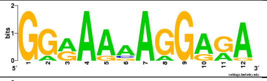	NFKB_Q6 (14)	1.10	0.111	0.296	SPI-1 (6)	1.45	0.401	0.665
b5h (15; 29)	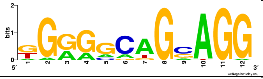	E47_01 (15)	0.99	0.011	0.059	SPI-1 (6)	1.74	0.580	0.670
b6h (6; 7)	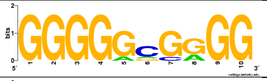	SP1_Q6 (13)	0.80	0.236	0.343	PPARgamma-RXRal (20)	1.86	0.136	0.365
b7h (6; 6)	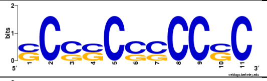	AP2_Q6_01 (13)	1.23	0.579	0.618	SP1 (10)	1.80	0.636	0.681
b8h (4; 6)	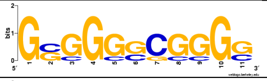	AP2_Q3 (16)	0.78	0.009	0.059	SP1 (10)	1.65	0.443	0.665
b9h (5; 6)	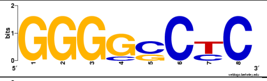	AP2_Q3 (16)	1.66	0.290	0.389	NF-kappaB (10)	2.48	0.556	0.670
b10h (6; 6)	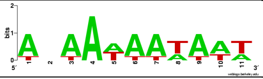	HP1SITEFACTOR_Q6 (12)	0.90	0.164	0.328	Pax-4 (30)	1.19	0.503	0.670
b11h (6; 6)	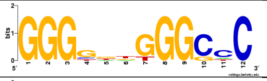	MZF1_02 (13)	0.88	0.081	0.259	MZF_5-13 (10)	0.67	0.005	0.075
b12h (4; 6)	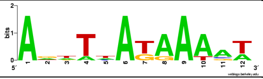	POU6F1_01 (11)	1.02	0.222	0.343	Pax-4 (30)	1.11	0.146	0.365
b13h (4; 6)	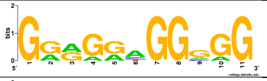	KROX_Q6 (14)	1.08	0.386	0.475	Gklf (10)	1.34	0.693	0.693
b14h (6; 6)	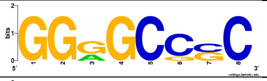	GC_01 (14)	1.51	0.226	0.343	p50 (11)	2.25	0.058	0.290
b15h (4; 6)	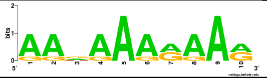	TBP_Q6 (7)	1.52	0.073	0.259	Gklf (10)	0.90	0.222	0.476
