# Supplementary material for: Inference of transcriptional regulation using gene expression data from the bovine and human genomes
Source: BMC Genomics. 2007 Aug 3;8:265. doi: 10.1186/1471-2164-8-265 (PMC1978505; doi:10.1186/1471-2164-8-265)
Supplement: Additional file 8 — Predicted motifs in the 15 bovine genes (group b). This file shows the 15 motifs detected in the promoter regions of the 15 bovine genes in group b. [file 1471-2164-8-265-S8.pdf]

Additional file 8 (Zadissa et al.)

Identified motifs detected in the promoter regions of the 15 bovine genes in group b. Sequence logos for the predicted motif and the expected TFBS are displayed, as are the calculated dissimilarity scores (S) and p-values (P) for each comparison (S_{cutoff} = 1.3, P_{cutoff} = 0.05). MAST analysis showed high similarity between the motifs, collapsing the 15 motifs in to six groups. These are: b1b; b2b, b4b, b5b, b6b, b7b, b10b, b12b and b14b; b3b; b8b; b9b, b11b and b13b; b15b.

Motif (Seqs; Sites)	Sequence logo	TRANSFAC hit	Score	p-value	Corrected p-value	JASPAR hit	Score	p-value	Corrected p-value
b1b (8; 19)	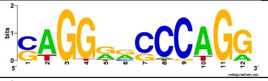	AREB6_01 (13)	1.15	0.056	0.132	AP2alpha (9)	2.24	0.360	0.540
b2b (6; 8)	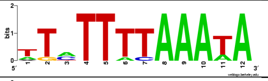	MEF2_Q6_01 (12)	0.92	0.072	0.132	Pax-4 (30)	1.46	0.021	0.173
b3b (11; 25)	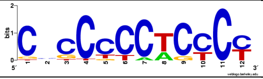	CACBINDINGPROTEIN_Q6 (9)	0.90	0.075	0.132	MZF_1-4 (6)	1.60	0.564	0.651
b4b (4; 6)	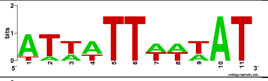	GATA3_03 (10)	0.95	0.213	0.246	Pax-4 (30)	1.35	0.023	0.173
b5b (5; 6)	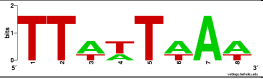	POU1F1_Q6 (10)	0.80	0.072	0.132	HNF-1 (14)	0.96	0.087	0.218
b6b (3; 6)	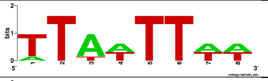	HNF1_01 (15)	1.00	0.088	0.132	HNF-1 (14)	0.90	0.069	0.207
b7b (3; 6)	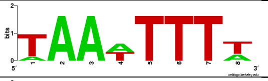	TBP_01 (8)	1.35	0.277	0.297	HNF-1 (14)	2.01	0.709	0.760
b8b (4; 6)	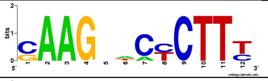	FOXO3_01 (14)	1.64	0.180	0.225	NRF-2 (10)	1.69	0.059	0.207
b9b (5; 6)	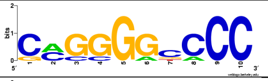	SP1_Q6 (13)	1.31	0.085	0.132	AP2alpha (9)	2.00	0.308	0.513
b10b (4; 6)	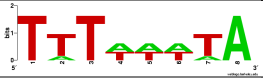	MEF2_Q6_01 (12)	0.55	0.010	0.132	MEF2 (10)	1.12	0.067	0.207
b11b (4; 6)	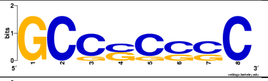	AP2_Q3 (16)	1.22	0.027	0.132	AP2alpha (9)	2.30	0.556	0.651
b12b (4; 6)	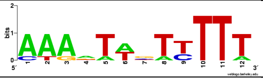	NFAT_Q6 (12)	1.34	0.362	0.362	HFH-2 (12)	2.18	0.790	0.790
b13b (5; 6)	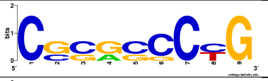	AP2_Q6_01 (13)	1.18	0.044	0.132	AP2alpha (9)	2.13	0.264	0.495
b14b (5; 6)	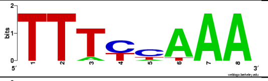	BARBIE_01 (15)	1.28	0.171	0.225	cEBP (12)	2.12	0.462	0.630
b15b (3; 6)	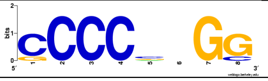	AP2_Q6_01 (13)	1.10	0.042	0.132	AP2alpha (9)	1.65	0.105	0.225
